# Magnetic Resonance Imaging of Nerve Roots in the Diagnosis of Chronic Inflammatory Demyelinating Polyneuropathy (CIDP) – A Systematic Review and Meta‐Analysis

**DOI:** 10.1111/ene.70612

**Published:** 2026-05-28

**Authors:** Karolina M. Gausmann, Rebecca A. May, Thorsten Sichtermann, Nicole M. Heussen, Jörg B. Schulz, Robert Brunkhorst

**Affiliations:** ^1^ Department of Neurology RWTH Aachen University Aachen Germany; ^2^ Department of Neuroradiology RWTH Aachen University Aachen Germany; ^3^ Institute of Medical Statistics RWTH Aachen University Aachen Germany; ^4^ Faculty of Medicine Sigmund Freud University Vienna Austria; ^5^ JARA‐BRAIN Institute Molecular Neuroscience and Neuroimaging Research Centre Juelich GmbH and RWTH Aachen University Aachen Germany

**Keywords:** chronic demyelinating, CIDP, MRI, nerve roots, polyradiculoneuropathy

## Abstract

**Background:**

Chronic inflammatory demyelinating polyneuropathy (CIDP) is a relevant differential diagnosis for progressive polyneuropathy, with effective treatment options available. In case of an ambiguous diagnosis, supportive tests like nerve imaging are recommended. However, MRI is considered of limited use by the current guidelines. This meta‐analysis provides an analysis of current data on morphological MRI features in CIDP.

**Methods:**

We searched databases in PubMed, Scopus, Web of Science, and Google Scholar and reported findings following PRISMA guidelines. We included prospective and retrospective case–control and cohort studies on qualitative or quantitative assessment of cervical and lumbar nerve roots in CIDP. The size and morphological appearance of nerve roots were compared between affected vs. non‐affected subjects.

**Results:**

We included 11 studies with qualitative and 15 with quantitative outcome measures. Odds ratios indicated hypertrophy, hyperintensity, and gadolinium enhancement in CIDP patients. Quantitative analysis confirmed larger nerve root diameters averaging at 5 mm in the cervical and 7 mm in the lumbosacral area. Few studies investigated the cross‐sectional area and volume of nerve roots, confirming larger values in CIDP patients.

**Conclusions:**

Plexus imaging provides valuable adjunctive support in the diagnosis of CIDP and our analysis revealed greater diagnostic significance for lumbar nerve roots. Yet its utility must be interpreted with nuance because of data heterogeneity and potential imaging mimics simulating CIDP features on MRI. Future advancements should focus on refining imaging techniques and developing quantitative metrics to enhance diagnostic specificity. The integration of imaging modalities with clinical and electrophysiological findings remains essential in diagnosis of CIDP.

## 1 Introduction

Chronic inflammatory demyelinating polyneuropathy (CIDP) is a rare form of acquired peripheral neuropathy with a prevalence of almost 3 per 100,000 persons [1, 2]. It is an immune‐mediated disease, targeting peripheral nerves and resulting in demyelination and axonal damage. The initial manifestation of the disease typically includes distal paraesthesia and proximal and distal para‐ or tetraparesis with diminished or absent reflexes. Clinical progression leads to gait disorder and impairment of daily life functions. CIDP is defined by a disease course of more than 8 weeks, but might be relapsing‐remitting [3]. Since it is potentially amenable to immune‐modulating treatment options that offer a chance of disability improvement, it represents a considerable differential diagnosis in clinical routine. In case of an asymmetric presentation, CIDP variants such as multifocal motor neuropathy (MMN) and multifocal sensory and motor neuropathy (MADSAM) must be distinguished from typical CIDP [4]. In the presence of pure sensory impairment, sensory CIDP must be considered and distinguished from chronic sensory inflammatory polyneuropathy (CISP) [3]. Variants of CIDP share signs of demyelination and response to immune therapy.

Diagnosis of CIDP is based on the combination of clinical appearance and electrophysiological findings. If clinical but not electrophysiological criteria are fulfilled, at least two supportive criteria are required for diagnosis. These include nerve ultrasound or MRI nerve imaging as well as objective treatment response as defined by established criteria in the guideline [3]. Conversely, CSF testing and nerve biopsy are not generally recommended [3]. Despite well‐defined diagnostic criteria, the wide range of clinical manifestations and various subtypes leads to frequent misdiagnosis, highlighting the importance of reconsidering the diagnosis throughout the clinical course. Furthermore, the effect of treatment might show up late and potentially require several treatment courses before improvement can be objectified. Inflammatory lesions mainly affect the spinal nerve roots, proximal nerve trunks and major nerve plexuses, areas, which are difficult to assess with standard neurophysiological methods. Deeper nerve structures such as the lumbar plexus, proximal leg nerves and specific segments of the brachial plexus are often inaccessible to ultrasound but remain evaluable with MRI. Therefore, MRI is increasingly used to detect alterations in the brachial and lumbosacral plexus with hypertrophy and T2‐hyperintensity of nerve roots being considered typical for CIDP [5]. Although contrast enhancement on MRI was considered a typical finding in CIDP in the past [6, 7, 8], it has been downplayed in the current guidelines [3]. A quantitative analysis of nerve roots is recommended, with a diameter of 5 mm serving as an indicative threshold [3]. However, objective and specific criteria for MRI evaluation are missing, so that recommendation of MRI is restricted to cases where other immune or hereditary demyelinating neuropathies are unlikely [3]. To ensure that sonographic or radiological abnormalities accurately support a CIDP diagnosis, it is essential to rule out any clinical or laboratory evidence pointing toward conditions that mimic CIDP, i.e. Charcot–Marie–Tooth disease, IgM paraproteinemic neuropathy, POEMS or amyloid neuropathy [9, 10, 11]. With this systematic review and meta‐analysis, we intended to describe and quantify MRI data which in parts resulted in the EAN guideline recommendations, providing a better understanding of the current knowledge and potential future markers.

## 2 Methods

This review was performed according to the PRISMA Guidelines 2020 (Preferred Reporting Items of Systematic Reviews and Meta‐Analyses, Table S1) [12]. Databases from PubMed, Scopus, Web of Science and Google Scholar were searched independently by two investigators in November 2022 using predefined search terms and without any publication date restriction. An updated search of the PubMed database, which provided the majority of relevant papers in the initial search, was conducted in 10/2025 and did not yield any new studies meeting the inclusion criteria. Search terms used were “polyradiculoneuropathy” or “chronic inflammatory demyelinating” and “magnetic resonance” or “MRI”. Relevant articles were identified on the basis of established inclusion and exclusion criteria. We excluded studies focussing on children, patients with Guillain‐Barré‐Syndrome or CIDP mimics (i.e. POEMS syndrome, paranodal/paraneoplastic polyneuropathy) and studies without MRI evaluation or with MRI of peripheral nerves.

**FIGURE 1:**
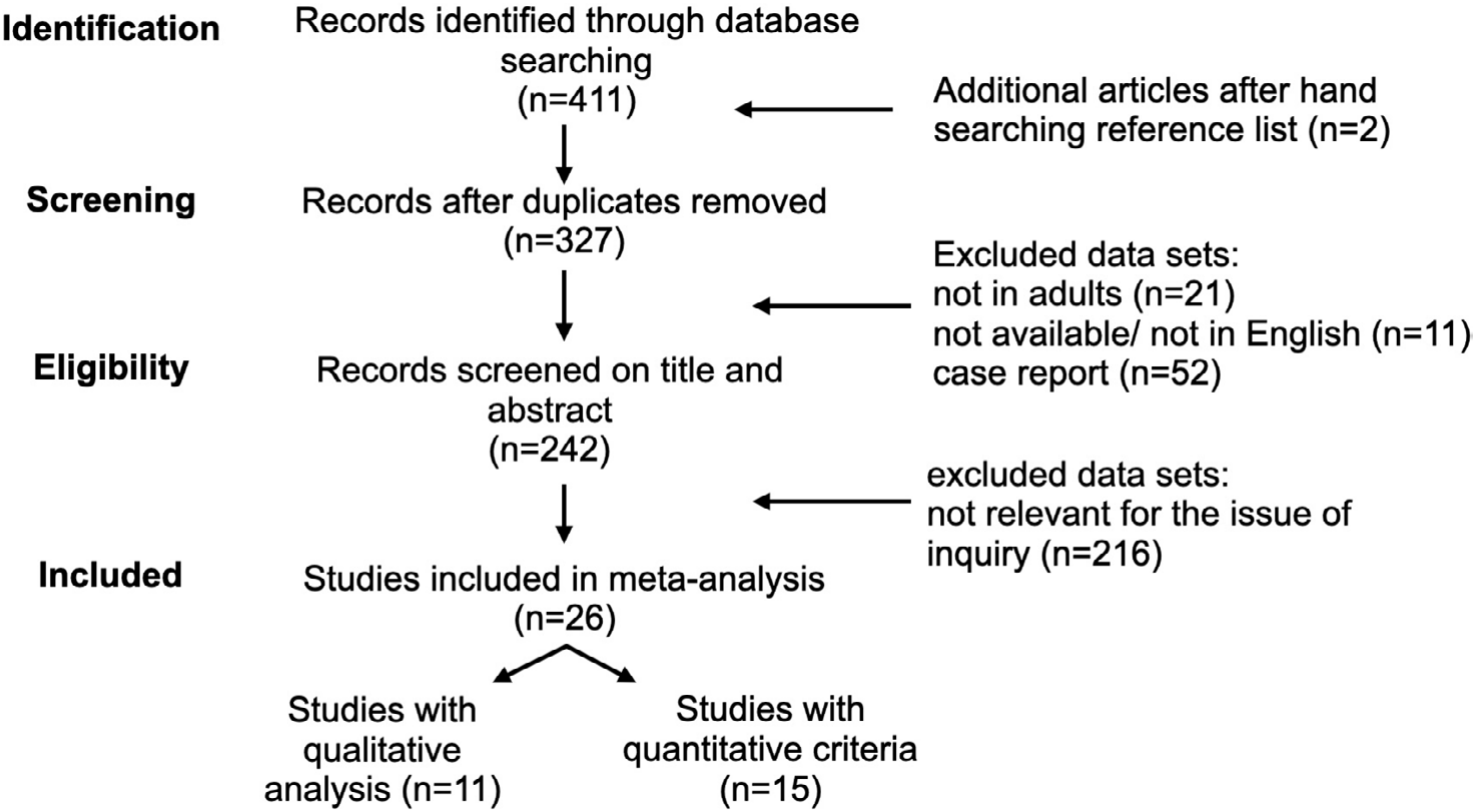
Selection process of eligible studies for the meta‐analysis.

We included all studies that reported on nerve roots of the cervicobrachial or lumbosacral MRI in patients with CIDP and met the following criteria (1) available in the English language and (2) as full text, (3) referring to adult subjects and (4) not restricted to case reports. Further selection was completed by screening titles and abstracts and in a next step the full text by relevance. We concentrated on studies examining nerve root thickness and appearance and excluded radiological parameters like diffusion properties or fractional anisotropy. Data from included publications were extracted into a MS Excel table. The selection process is depicted in Figure 1. On the basis of the type of MRI evaluation, studies were classified into quantitative and qualitative analysis. Among the studies with quantitative analysis, pooled effect sizes were determined depending on the location of measurement in relation to the ganglion. Additionally, we calculated a pooled mean per anatomic region. In studies with qualitative outcome measures we calculated odds ratios for the presence of “abnormalities” and hypertrophy, hyperintensity and contrast enhancement, when specified.

For statistical analysis we used GraphPad Prism 8.0 (GraphPad Software, San Diego, CA, USA) and RevMan 5.4. Unless otherwise specified, values are expressed as mean ± standard deviation. Pooled means and 95% confidence limits for affected and non‐affected groups were calculated using the generic inverse variance approach in RevMan. RevMan was also used to calculate pooled effect size estimates as standardised mean difference (SMD) and corresponding 95% confidence limits using a random effects model and measures of heterogeneity (*I* ^2^, Chi^2^, Tau^2^). Subgroup analysis on the basis of MRI field strength was performed to identify causes of heterogeneity. Odds ratios were calculated using the Peto method. The risk of bias assessment was carried out using the Newcastle‐Ottawa Scale for case–control and cohort studies according to the study design. Potential of bias was assessed for each study with guiding questions in three areas selection, comparability and exposure or outcome depending on the type of study. Within each area, stars were allocated to the guiding questions. A high number of stars corresponds to a low risk of bias. Additionally, GRADE guidelines were used to assess quality of evidence [13].

### 2.1 Standard Protocol Approvals, Registrations and Patient Consents

This study covers only published patient data; therefore, institutional ethics review was waived.

## 3 Results

### 3.1 Population Characteristics

A total of 411 studies were identified via database research, 2 were manually added and 85 duplicates were removed. After applying inclusion and exclusion criteria, a total of 26 articles were selected for data extraction. Articles were categorised in qualitative (*n* = 11) and quantitative (*n* = 15) analysis on the basis of the type of MRI processing (Figure 1). 10 of the 15 quantitative studies measured the diameter, 5 calculated the cross‐sectional area of nerve roots and 1 study carried out volumetry. In all 26 studies, radiologists analysing MRI scans were blinded to the diagnosis.

The meta‐analysis of these studies refers to a total cohort of 516 CIDP patients and 527 controls, defined as non‐CIDP. 198 CIDP patients and 245 controls were assessed by qualitative approach studies, and MRIs of 318 CIDP patients and 282 controls were analysed quantitatively (Table 1). The single cohort sizes varied between 5 and 50 CIDP patients and 0–174 controls. In 14 studies, the control group consisted of healthy subjects, in eight studies of other neurological diseases such as motoneuron disease, diabetic polyneuropathy or conditions requiring cervical MRI, and in one study, the control group consisted of both healthy subjects and other neurological diseases [14]. In four studies, there was no non‐CIDP group [15, 16, 17, 18]. Eight authors investigated typical CIDP and CIDP variants [15, 18, 19, 20, 21, 22, 23, 24]. As the publication date was not restricted in our search, included studies dated from 1997 to 11/2022, and the diagnosis of CIDP was based on the prevailing EFNS/PNS criteria at the time of publication. An updated search during the revision process identified no new relevant studies. The investigated CIDP cohorts consisted of both pretreated and untreated patients with a variable disease duration.

**TABLE 1:** Demographic characteristics of the control and the CIDP cohort.

	Control	CIDP	CIDP variant
*n*	527	516	138
Quantitative	282	318	41
Qualitative	245	198	92
Age (years)	50 ± 10	51 ± 8	48 ± 5
Male (%)	66%	65%	84%

*Note:* Data on age and/or sex was not provided by every author. Therefore, calculation of the mean is based on (a) 17, (b) 24, (c) 7, (d) 17, (e) 23 and (f) 6 studies.

Characteristics of the investigated population are depicted in Table 1. Because of the study design, demographic data could not be deduced from two studies [24, 25]. Three authors did not report the age and the fraction of men in the control group [19, 20, 26] whereof one author also did not provide data on the fraction of men in the CIDP group [26]. Furthermore, seven authors did not provide the mean but the median age.

### 3.2 Technical Features

Magnetic resonance imaging was performed on 3 T MRI devices (*n* = 16) or on 1.5 T (*n* = 11), less frequently on 0.5 T devices (*n* = 3), and once on a 1 T device. Five studies used two different devices. Gadolinium as a contrast agent was administered in 12 studies. Where indicated, a concentration of 0.1 mL/kg was applied. Most authors performed T2‐weighted STIR and T1‐weighted sequences. DWI was used additionally by three authors [22, 25, 27]. Several authors used special MRI techniques like 3D SHINKEI [28, 29, 30], DWIBS [31], 3D SPACE [21, 22, 24, 32, 33], 3D SPIR [23] and MP‐RAGE [34]. Accordingly, echo time, repetition time, slice thickness, and gap sizes varied substantially.

### 3.3 Measurement Site

Nerve root diameters were measured distal to (*n* = 6 [22, 23, 30, 32, 33, 34]) or at the dorsal root ganglion (*n* = 3 [28, 29, 30]), alternatively at the foraminal outlet (*n* = 5 [14, 28, 29, 34, 35]) or intraforaminally (*n* = 1 [34]) (Figure S1). In five studies, measurement was conducted at multiple locations: Hiwatashi et al. measured the size of the ganglion and the roots (2017, 2019) respectively of the spinal nerves (2018) without further specification of the exact position [28, 29, 30]. Van Rosmalen et al. compared the diameter just next to the ganglion and 1 cm distal of it [23] and Shah et al. measured before, at and 2 cm behind the foraminal outlet [34], which corresponded to a localisation behind the ganglion.

### 3.4 Nerve Root Diameter

All but two quantitative studies [22, 35] showed significantly larger nerve roots in CIDP patients. In the cervicobrachial region, six authors provided values per nerve root (for C8, *n* = 5); otherwise, the diameter was given as an average of the whole section. In CIDP patients, the nerve root size increased in caudal direction with a maximum at C7. In healthy controls, the diameter of C5 was smaller than that of C6‐8. The difference between nerve root sizes of CIDP and control subjects was largest for nerve roots C7 and C8.

**FIGURE 2:**
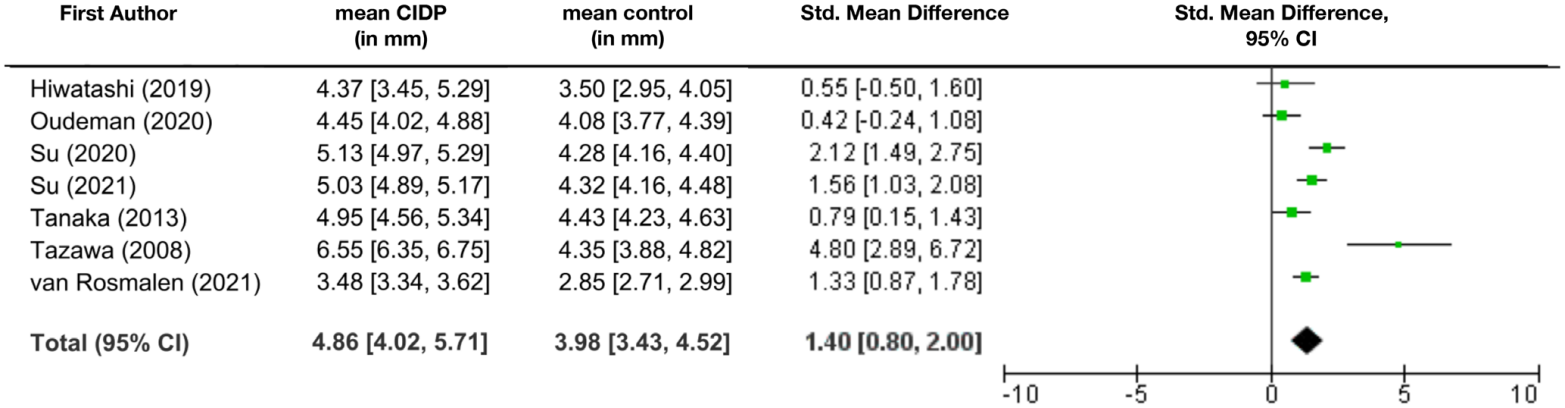
Mean diameters of cervical nerve roots (in mm) with 95% CI and standard mean difference. Mean diameter across all studies was calculated as weighted arithmetic mean.

The mean cervical diameter from all studies was 4 mm [95% CI: 3.4 mm; 4.5 mm] for non‐CIDP patients and 4.9 mm [95% CI: 4 mm; 5.7 mm] in the CIDP group (Figure 2). Calculation included values proximal and distal to the ganglion, whereas data from measurement *at* the ganglion was omitted to avoid bias since the ganglion is physiologically thicker than the nerve. On average, the root diameter of CIDP patients was significantly larger than in non‐CIDP patients (SMD 1.4 [95% CI: 0.8; 2.0], Figure 2). Differences in diameter varied slightly depending on the placement of measurement, in CIDP patients tending to be larger before than behind the ganglion (SMD 1.81 [0; 3.61] for proximal and 1.36 [0.76; 1.97] for distal measurement).

**FIGURE 3:**
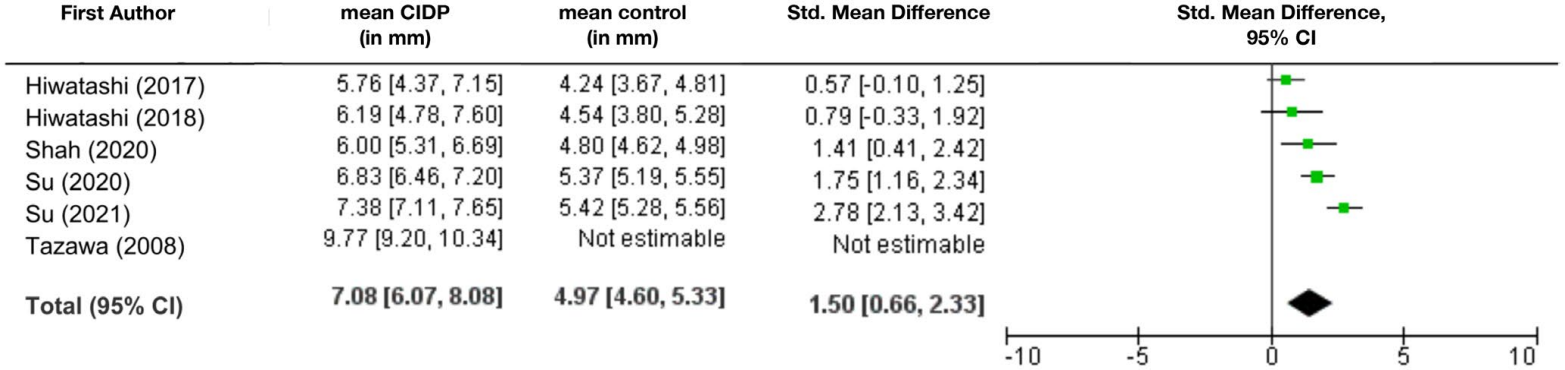
Mean diameters of lumbar nerve roots (in mm) with 95% CI and standard mean difference. Mean diameter across all studies was calculated as weighted arithmetic mean.

In the lumbosacral area, CIDP patients also showed distinct nerve hypertrophy compared to non‐CIDP patients (SMD 1.5 [95% CI: 0.66; 2.33], Figure 3). The difference was even more significant when measuring the distal ganglion (SMD 0.92 [95% CI: 0.1; 1.73] proximal vs. 2.4 [95% CI: 1.22; 3.58] distal the ganglion). The average nerve root size was 5 mm [95% CI: 4.6 mm; 5.3 mm] in the control group and 7.1 mm [95% CI: 6.1 mm; 8.1 mm] in CIDP patients. A differentiated analysis of individual nerve roots was not possible in the lumbosacral area, since only three authors provided data on single nerve roots.

Among the studies with quantitative outcome measures, one study utilised contrast agent; however, no contrast enhancement was detected in any of the subjects [15].

Variability across studies was high (*I* ^2^ > 80%, Tau^2^ 0.14–1.4, Chi^2^ with *p* < 0.1). In the cervicobrachial area, diameters reported by van Rosmalen et al. appear to be a low outlier, whereas diameters calculated by Tazawa et al. stand out as a high outlier in the cervicobrachial and lumbosacral region. Strikingly, the population studied by van Rosmalen was older (mean age 63.8 years) than the population investigated by Tazawa et al. (mean age 38.9 years), so that one might suppose an age‐dependent effect because of atrophy of nerve roots [36]. However, the cohort of Oudeman et al. showed larger nerve roots than van Rosmalen's, despite comparable average age. Furthermore, the overall studies examined in our meta‐analysis did not indicate any age‐dependency either [15, 34]. Subgroup analysis for the cervicobrachial region on the basis of MRI field strength demonstrated consistently high heterogeneity and revealed no significant differences between the subgroups, suggesting that field strength is unlikely to account for the observed variability (Figure S2). In the lumbosacral region, subgroup analysis was considered redundant as the only study using a 1.5 T device (Tazawa et al.) was excluded from the SMD calculation because of the absence of control group data.

### 3.5 Cross‐Sectional Area and Volumetry

**FIGURE 4:**
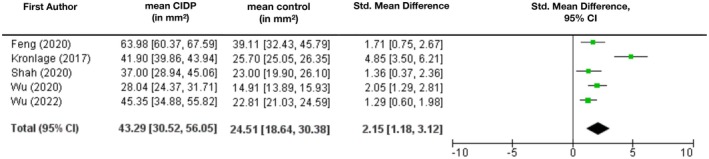
Mean cross‐sectional area (CSA) of lumbar nerve roots in mm^2^ with 95% CI and standard mean difference. Mean diameter across all studies was calculated as weighted arithmetic mean.

Consistent with the findings on the diameter, the cross‐sectional area was also reported to be larger in CIDP patients. Five authors provided data on lumbosacral nerve root area [21, 34, 37, 38, 39]. On average, the cross‐sectional area (CSA) amounted to 43.29 mm^2^ [95% CI: 30.52 mm^2^; 56.05 mm^2^] compared to 24.51 mm^2^ [95% CI: 18.64 mm^2^; 30.38 mm^2^] in healthy patients (SMD 2.15 [95% CI: 1.18; 3.12], Figure 4). The area of cervical nerve roots was investigated by only one author and likewise proved to be larger in CIDP patients [21]. Volumetric studies conducted by Ishikawa et al. showed similar results: They reported mean values for the volume adjusted to body surface of 19.46 ± 12.28 cm^3^/m^2^ for CIDP and 10.14 ± 2.18 cm^3^/m^2^ for control patients in the cervicobrachial area, respectively 23.46 ± 13.53 cm^3^/m^2^ (CIDP) vs. 6.9 ± 1.69 cm^3^/m^2^ (control) in the lumbosacral area [31].

### 3.6 Qualitative Outcome Analysis

Among the qualitative measurements, four studies evaluated cervical nerve roots, two investigated the lumbar nerve roots and/or cauda equina and five considered both areas. Radiologists were blinded to the diagnosis and assessed the appearance of nerve roots according to their experience. In some studies, findings were reported as “abnormalities”, whereas others provided more detailed evaluation of nerve hypertrophy, signal hyperintensity and contrast enhancement. Most authors assessed “enhancement” by comparing images before and after gadolinium administration. In three studies, there were no further details on the definition of contrast uptake [16, 20, 25]. The used MRI scanners were mainly 1.5 T devices (*n* = 8), followed by 3 T (*n* = 3), 0.5 T (*n* = 2) and 1 T (*n* = 1). In four studies, two different devices were used. Since results were not stratified by field strength, performing a subgroup analysis for this parameter was not possible.

Furthermore, because of different study designs, comparative analysis among the studies was not feasible. Abnormalities were found in 50% of CIDP patients, hypertrophy and high intensity signal in T2‐ or STIR. Three authors used a prospective design where the diagnosis of patients fulfilling or not fulfilling criteria for definite CIDP was made after MRI. Patients with atypical presentation and electrophysiology at baseline had abnormal radiological findings in 60% [24] and in 44% [25], and a diagnosis of typical CIDP or CIDP variant was made in 63% and 65% after MRI. In CIDP patients, Lozeron et al. described hypertrophy and hyperintensity in 40% and 46%, respectively. The published data from Fargeot et al. do not allow conclusions about the frequency of abnormalities in CIDP variants. In the study of Jomier et al., where patients were followed up over a 2‐year observation period, MRI was not considered beneficial, as it was abnormal only in five of the patients later diagnosed with CIDP [40]. The remaining eight studies with a qualitative approach performed a retrospective evaluation of MRI in CIDP patients and a non‐CIDP group consisting of healthy controls (*n* = 1 [27]) or other neurological diseases (*n* = 3 [19, 20, 26]). In four studies, there was no non‐CIDP group [15, 16, 17, 18]. Four authors examined CIDP variants in addition to typical CIDP [15, 19, 20, 26]. Pooled data from the eight retrospective studies and one prospective [40] showed abnormal MRI in 59%, hypertrophy in 53%, and hyperintensity in 59% of CIDP patients. CIDP variants showed abnormalities less frequently (hypertrophy in 40% and hyperintensity in 32%). Gadolinium uptake was examined in seven studies, of which three were with CIDP variants. Enhancement was found in three studies and on average in 23% of CIDP patients, and none of the CIDP variants. However, absolute numbers were small. Adachi et al. [27] and Duggins et al. found enhancement in 3 of 10 (30%), respectively, 6 of 11 (45%) patients. Midroni et al. [26] described enhancement in 11/16 (69%) cases. In non‐CIDP patients, abnormalities were only described by Van Es et al. and concerned patients with radiation fibrosis and posttraumatic conditions who showed increased signal intensity or patients with tumour, where contrast uptake correlated with tumour mass [20].

**FIGURE 5:**
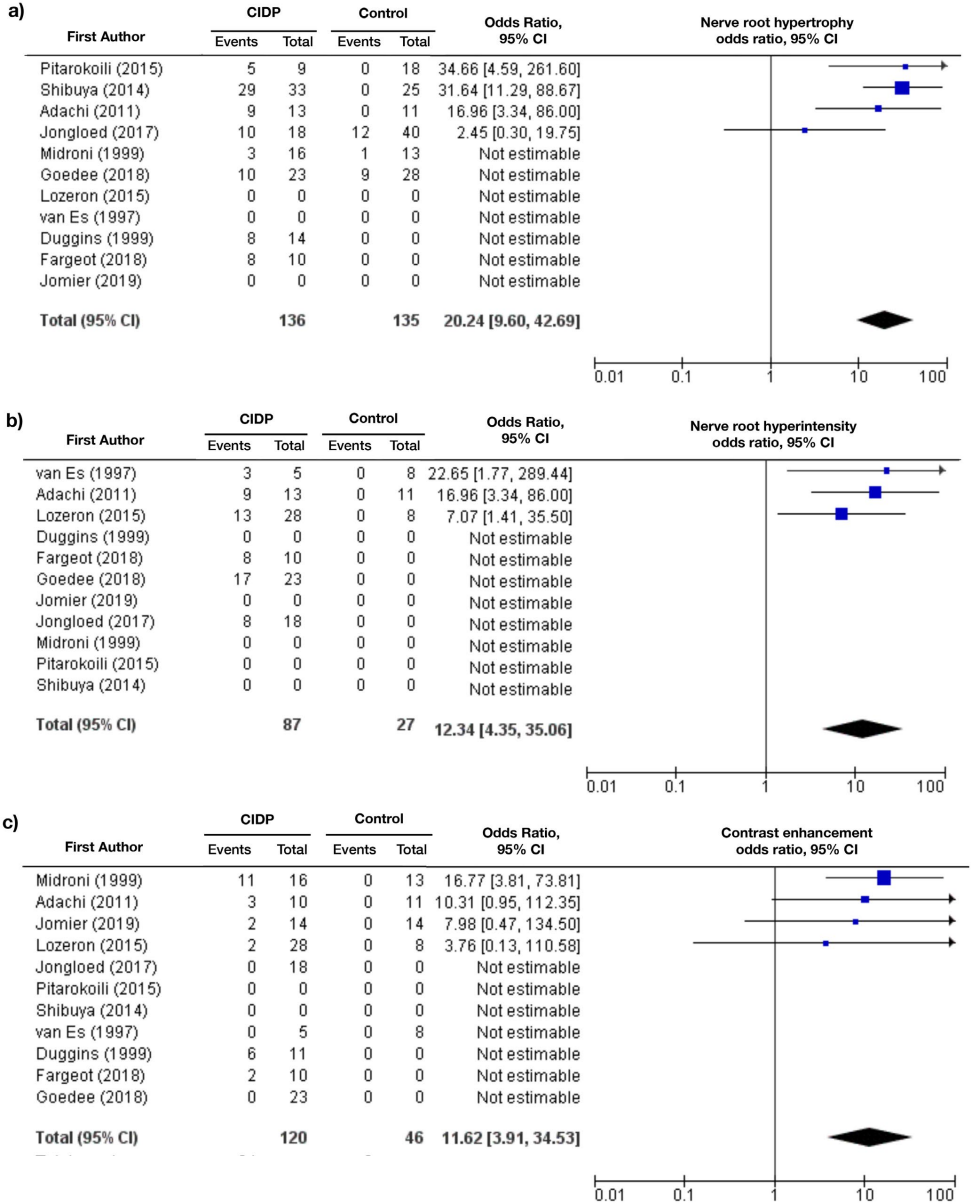
Forest Plot of Peto odds ratios for (a) hypertrophy, (b) hyperintensity on T2‐ and STIR‐weighting and (c) contrast enhancement of nerve roots in CIDP patients and controls. The number of events and population size are given in absolute numbers. Entries with a total of “0” indicate that the outcome was not considered by the study or that there was no control group.

Because of the variability of study designs (i.e. indication of results as number of plexuses rather than number of patients or application of contrast agent only in a part of the studied population), only a few studies were taken into consideration, calculating odds ratios. Figure 5 depicts conclusions drawn from qualitative analysis. Hypertrophy and hyperintensity were more frequent in CIDP patients (OR 20.24 [9.6; 42.69], Figure 5a), respectively 12.34 [4.35; 35.06] (Figure 5b). The odds ratio for contrast enhancement was 11.62 [3.91: 34.53] (Figure 5c) on the basis of four studies. These results have to be considered with caution seen the low prevalence and small sample sizes as described above, and the low prevalence in the control group, leading to a high OR.

### 3.7 Correlation With Clinical and Technical Characteristics

Across studies, the distribution pattern of abnormalities correlated with clinically affected regions and was symmetrical in CIDP and asymmetrical in CIDP variants [18, 19, 20, 24, 25, 32]. Involvement of the brachial plexus was observed even in patients presenting with isolated lower limb weakness [18]. Except for one study that found a correlation between hypertrophy and disease duration [16], most authors reported no association between the extent of changes and disease duration [18, 32, 34, 40] or previous treatment [14, 15, 16, 18, 25, 27, 40]. Similarly, the magnetic field strength was not reported to influence measured nerve root thickness [25].

### 3.8 Risk of Bias Analysis

**FIGURE 6:**
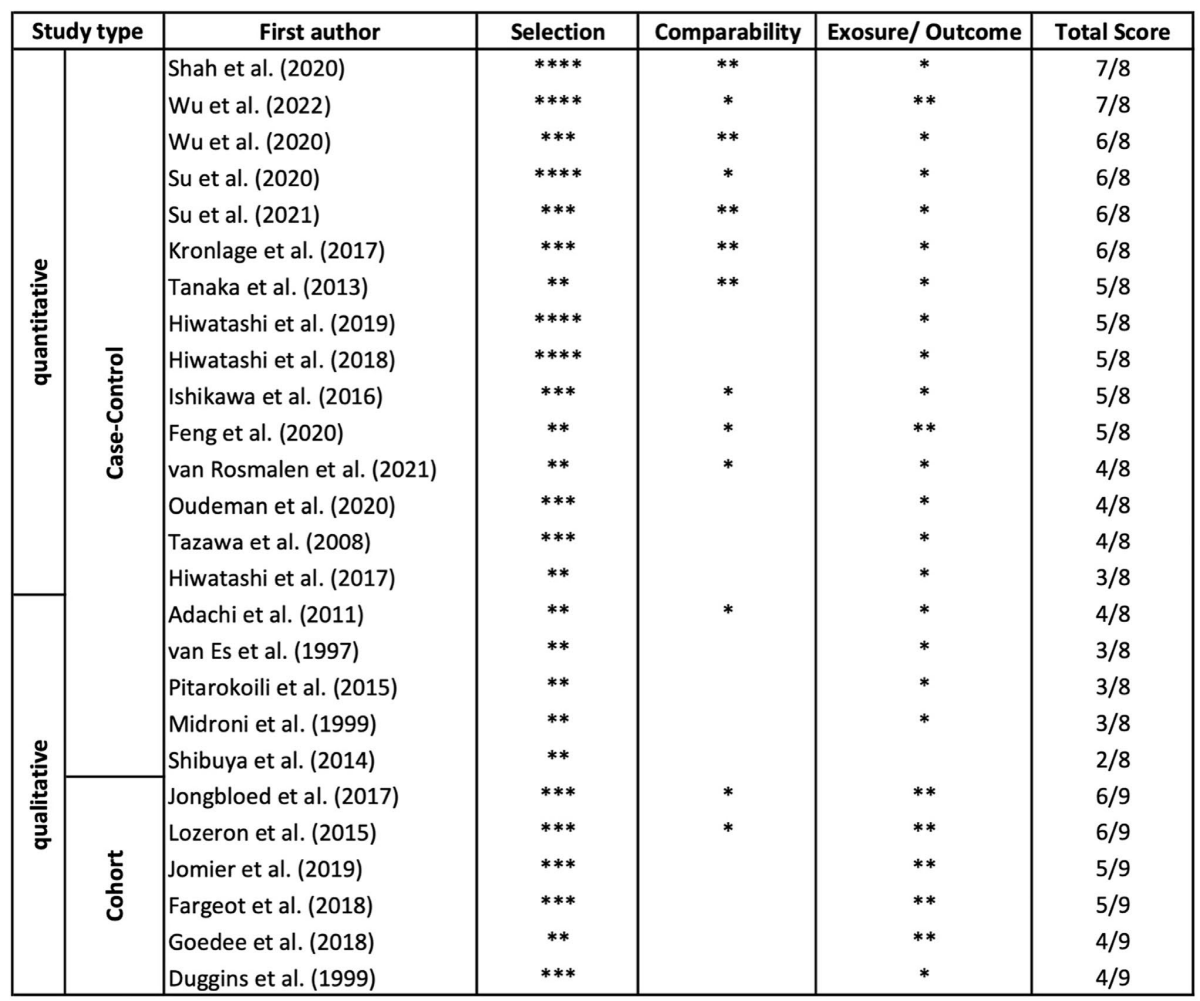
Newcastle‐ Ottawa Scale for Risk of Bias analysis. The risk of bias was assessed in three areas: Selection, comparability and exposure or outcome, depending on the type of study. The higher the number of allocated stars, the lower the potential of bias. The maximum score was 9 for cohort and 8 for case–control studies since the “non‐response rate did not apply for any of the studies included”.

The risk of bias was assessed using the Newcastle‐Ottawa Scale [41] for case–control and cohort studies (Figure 6) and the certainty of evidence was subsequently evaluated according to the GRADE guidelines (Figure S3). The mean risk of bias of the qualitative case–control studies was 3/8 (range 2–4) and 5/8 (range 3–7) for quantitative studies. Cohort studies showed a mean risk of bias of 5/9 (range 4–6). Since no intervention or treatment took place, the “non‐response rate” for case–control studies was not applicable, and the maximum number of stars was 8 in this category. The assessment tools revealed only a moderate (for quantitative) or low (for qualitative) certainty of evidence, which was mainly attributable to variations in study protocols and inhomogeneous control groups, as well as missing data from control groups. An additional potential confounder is that studies refer to the CIDP criteria of the time, and more CIDP‐mimics are known by now, i.e. paranodopathies, which could have caused false positive results in the past, as some authors described massive thickening of nerve cords [16].

Since the number of included studies was insufficient to permit a robust evaluation of potential publication bias, we did not consider a funnel plot analysis to be methodologically appropriate (Figure S4).

## 4 Discussion

In this meta‐analysis, we evaluated 26 studies reporting on MRI‐based quantitative and qualitative features of nerve roots in CIDP.

The quantitative comparison of nerve root diameters indicates significantly larger values in CIDP patients as opposed to healthy controls or other neurological diseases without demyelinating pathology. The difference is particularly prominent when measuring behind the ganglion in the lumbosacral area (SMD 2.4 [95% CI: 1.22; 3.58]). Radiological changes occurred in clinically affected regions and were consistent with the pathophysiology (symmetrical in CIDP, asymmetrical in CIDP variants). No correlation with disease duration or treatment status was documented.

The current EAN guidelines emphasise the paucity of data on cut‐off values for evaluating nerve root sizes on MRI. A cut‐off value of 5 mm is suggested for measuring cervical and lumbar nerve roots [3]. In the literature, this cut‐off is described by Tazawa et al. for cervicobrachial nerve roots [14] and by Midroni et al. for the lumbosacral region [42]. Our meta‐analysis indicates that the average cervical root diameter in CIDP patients matches the 5 mm suggested by the guideline. However, the confidence interval ranging from 4.0 to 5.7 mm indicates that a 5 mm cut‐off might overlook some cases of CIDP. Also, distinction to control subjects might be challenging in the range from 4 to 4.5 mm. In the case of lumbosacral nerve roots, our data reveal larger nerve roots than suggested by the guideline, with an average of 7 mm. In view of the non‐overlapping confidence intervals, distinction from healthy controls seems possible in this area. In addition, Chaves et al. showed diameters of about 4.5 mm in healthy subjects [43]. However, we show here that the available data so far is too heterogeneous to determine reliable thresholds. Different cut‐offs for the cervicobrachial and lumbosacral region seem useful but require further validation in larger population sizes. Also, the impact on individual nerve roots and size variability should be addressed in future research, since in CIDP, a single nerve root can be more affected than others [25]. The joint analysis of all nerve roots within a region could have contributed to the observed variability in our study.

Our qualitative analysis revealed that hypertrophy and hyperintensity in T2‐ or STIR‐weighting are potential parameters to distinguish CIDP from healthy controls or other neurological conditions without demyelinating pathology. However, the differentiation between CIDP and variants [19] cannot be performed reliably with the described markers. Furthermore, CIDP mimics may present with similar findings in MRI, i.e., hypertrophy of nerve roots was also observed in Charcot–Marie‐Tooth disease [19, 26].

As a consequence of an inflammatory breakdown of the blood‐nerve barrier [44], one might expect to find Gadolinium uptake in the case of CIDP. However, as we show here, current data do not support this hypothesis. Only one author described contrast uptake in most of the investigated CIDP patients [26]. However, prominent nerve root enlargement is described here, which might suggest differential diagnoses (i.e. paranodopathies) from today's point of view.

Current evidence is limited by small sample sizes, especially of control groups, and inhomogeneous study designs with variations in MRI protocols, slice thickness, location of measurement, and analytical methods. Data on inter‐ and intra‐rater reliability are contradictory [22, 23, 45] and standardised protocols and unsupervised machine learning or artificial intelligence could enhance the reliability and consistency of measurements. Quantitative measures should be favoured to enhance reproducibility. To reduce variability in nerve root measurements a highly standardised protocol is essential, encompassing technical features of MRI (plane imaging, gap, sequence, slice thickness and signal averaging) and standardised definition of the site of measurement. Additionally, more granular reference values are needed, which are specific to the disease, site of measurement, and population characteristics like age. Contemporary MRI techniques now offer a more precise insight into nerve root integrity. Structural alterations can be more accurately assessed using tract‐based spatial statistics (TBSS) or diffusion tensor imaging (DTI), with diffusivity allowing identification of axonal and myelin damage and possibly correlating with disease duration [22, 27, 33]. Isotropic voxel acquisitions might be used to guarantee comparability of imaging protocols across studies.

Another promising approach for future research could also be the use of other contrast agents. Gadolinium uptake was not found in studies on peripheral nerve injury [46, 47], despite extensive destruction and active Wallerian degeneration. At the same time, studies on demyelinating inflammatory disease in the central nervous system indicate that a defective blood–brain barrier does not necessarily colocalize with macrophage invasion [44]. On the basis of this, some authors claim that in multiple sclerosis, the lesion burden is underestimated by application of Gadolinium, and they recommend the use of more sensitive contrast agents, such as Gadofluorine, or the use of small superparamagnetic iron oxide particles, which are phagocytosed by macrophages after intravenous application [44, 48]. In the peripheral nervous system, Gadofluorine has also been found in the context of Wallerian degeneration in the form of phagocytosed myelin debris in macrophages [48]. Thus, the use of other contrast agents could be a possible approach to detect inflammation activity in CIDP more precisely.

Our data suggests that nerve root enlargement and T2‐hyperintensity in MRI scans are indicative of CIDP, whereas contrast enhancement should not be considered a reliable parameter so far. A recommended cut‐off value of 5 mm appears to be plausible only for the cervical area, as lumbar nerve roots tend to be larger in CIDP patients. However, to refine these thresholds, additional studies are necessary to define standardised reference values that account for age‐related changes, disease‐specific characteristics and the specific anatomical site of measurement. As differential diagnoses mimicking CIDP can result in comparable MRI findings, the assessment of nerve roots must always be interpreted within the clinical context [3].

## Author Contributions

**Thorsten Sichtermann:** writing – review and editing. **Robert Brunkhorst:** conceptualization, investigation, methodology, writing – review and editing, supervision, project administration, formal analysis. **Rebecca A. May:** writing – review and editing. **Nicole M. Heussen:** methodology, writing – review and editing. **Karolina M. Gausmann:** conceptualization, investigation, writing – original draft, writing – review and editing, methodology, visualization, validation, formal analysis, project administration, data curation. **Jörg B. Schulz:** writing – review and editing.

## Funding

This work was supported by the European Union, as part of the European Rare Disease Research Alliance (ERDERA), GA no. 101156595, funded under call HORIZON‐HLTH‐2023‐DISEASE‐07.

## Conflicts of Interest

The authors declare no conflicts of interest.

## Supporting information


**Figure S1:** Outcome parameters and position of measurement in quantitative studies. Most investigators focussed on nerve root diameter distal the ganglion, whereas the exact location varied: Oudeman (2020) measured directly after the ganglion, van Rosmalen (2020) compared the diameters directly behind and 1 cm distal to the ganglion, and Su also measured 1 cm distal to the ganglion. Shah explored nerve roots intraforaminally, at the foraminal outlet and 2 cm distal the outlet. Hiwatashi investigated lumbar ganglia and spinal nerves (2018) respectively nerve roots (2017, 2019) without further specification of the exact measurement placement.

**Figure S2:** Subgroup analysis of studies evaluating cervical nerve root diameter by MRI field strength.

**Figure S3:** Assessment of the quality of evidence according to GRADE‐criteria.

**Figure S4:** Funnel plots assessing publication bias of included studies. Funnel plots provided for reference only.

**Table S1:** PRISMA 2020 checklist indicating the location of reported items within the manuscript.

## Data Availability

The first author has full access to the data used in the analysis. Data not provided in the article because of space limitations may be shared at the request of any qualified investigator for purposes of replicating procedures and results.
