# Supplementary material for: Magnetic Resonance Imaging of Nerve Roots in the Diagnosis of Chronic Inflammatory Demyelinating Polyneuropathy (CIDP) – A Systematic Review and Meta‐Analysis
Source: Eur J Neurol. 2026 May 28;33(5):e70612. doi: 10.1111/ene.70612 (PMC13240206; doi:10.1111/ene.70612)
Supplement: Supplementary file 1 — Figure S1: Outcome parameters and position of measurement in quantitative studies. Most investigators focussed on nerve root diameter distal the ganglion, whereas the exact location varied: Oudeman (2020) measured directly after the ganglion, van Rosmalen (2020) compared the diameters directly behind and 1 cm distal to the ganglion, and Su also measured 1 cm distal to the ganglion. Shah explored nerve roots intraforaminally, at the foraminal outlet and 2 cm distal the outlet. Hiwatashi investigated lumbar ganglia and spinal nerves (2018) respectively nerve roots (2017, 2019) without further specification of the exact measurement placement. [file ENE-33-e70612-s005.docx]

Supplementary Figure 1

| **Measurement Site** | **First Author** |
| --- | --- |
| intraforaminal | Shah (2020) |
| foraminal outlet | Tazawa (2008) |
|  | Tanaka (2013) |
|  | Hiwatashi (2017) |
|  | Hiwatashi (2019) |
|  | Shah (2020) |
| at the ganglion | Hiwatashi (2017) |
|  | Hiwatashi (2018) |
|  | Hiwatashi (2019) |
| distal the ganglion | Hiwatashi (2018) |
|  | Oudeman (2020) |
|  | Shah (2020) |
|  | Su (2020) |
|  | Su (2021) |
|  | van Rosmalen (2021) |
