# Supplementary figures and images for: Magnetic Resonance Imaging of Nerve Roots in the Diagnosis of Chronic Inflammatory Demyelinating Polyneuropathy (CIDP) – A Systematic Review and Meta‐Analysis
Source: Eur J Neurol. 2026 May 28;33(5):e70612. doi: 10.1111/ene.70612 (PMC13240206; doi:10.1111/ene.70612)

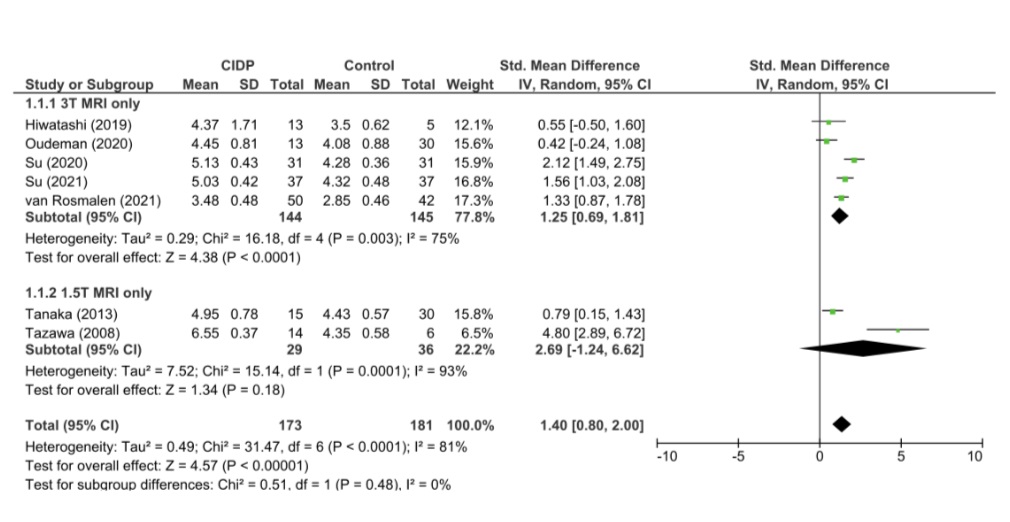

Supplement: Supplementary file 2 — Figure S2: Subgroup analysis of studies evaluating cervical nerve root diameter by MRI field strength. [file ENE-33-e70612-s003.jpg]
