# Supplementary material for: Magnetic Resonance Imaging of Nerve Roots in the Diagnosis of Chronic Inflammatory Demyelinating Polyneuropathy (CIDP) – A Systematic Review and Meta‐Analysis
Source: Eur J Neurol. 2026 May 28;33(5):e70612. doi: 10.1111/ene.70612 (PMC13240206; doi:10.1111/ene.70612)
Supplement: Supplementary file 3 — Figure S3: Assessment of the quality of evidence according to GRADE‐criteria. [file ENE-33-e70612-s001.pdf]

Question: Cervicobrachial and lumbosacral nerve root diameter in CIDP vs. controls as measured on MRI scans

Certainty assessment							Impact	Certainty
№ of studies	Study design	Risk of bias	Inconsistency	Indirectness	Imprecision	Other considerations		
7	non-randomised studies	not serious	serious[a]	not serious	serious[b]	strong association	SMD 1.40 [95% CI: 0.8, 2.00]	⨁⨁⨁◯ Moderate[a,b]
6	non-randomised studies	not serious	serious[a]	not serious	serious[b]	strong association	SMD 1.50 [95% CI: 0.66, 2.33]	⨁⨁⨁◯ Moderate[a,b]
5	non-randomised studies	not serious	serious[a]	not serious	serious[b]	strong association	SMD 2.15 [95% CI: 1.18, 3.12]	⨁⨁⨁◯ Moderate[a,b]

CI: confidence interval, **SMD**: standard mean difference

Explanations

a. Visual inconsistency and statistical analysis showing heterogeneity, however effect consistent in direction

b. Some imprecision due to wide confidence intervals and small sample sizes

Question: Should hyperintensity be used to diagnose CIDP in MRI?

Outcome	№ of studies (№ of patients)	Study design	Factors that may decrease certainty of evidence					Test accuracy CoE
			Risk of bias	Indirectness	Inconsistency	Imprecision	Publication bias	
**True positives** (patients with CIDP)	5 studies 69 patients	cohort & case-control type studies	serious[a]	not serious	serious[b]	very serious[c]	strong association	⨁◯◯◯ Very low [a,b,c]
**False negatives** (patients incorrectly classified as not having CIDP)								
**True negatives** (patients without CIDP)	4 studies 87 patients	cohort & case-control type studies	serious[a]	not serious	serious[b]	very serious[c]	strong association	⨁◯◯◯ Very low [a,b,c]
**False positives** (patients incorrectly classified as having CIDP)								

Explanations

a. missing data of control groups, in parts no gender- and age-adjustment, inhomogeneous control groups, heterogeneous study designs

b. highly variable effects sizes

c. use of different field strengths in some studies while results were not attributable to field strength, data based on few studies with small sample sizes, wide confidence intervals of individual studies

Question: Should hypertrophy be used to diagnose CIDP in MRI?

Outcome	№ of studies (№ of patients)	Study design	Factors that may decrease certainty of evidence					Test accuracy CoE
			Risk of bias	Indirectness	Inconsistency	Imprecision	Publication bias	
**True positives** (patients with CIDP)	11 studies 136 patients	cohort & case-control type studies	serious[a]	not serious	serious[b]	very serious[c]	strong association	⊕◯◯◯ Very low [a,b,c]
**False negatives** (patients incorrectly classified as not having CIDP)								
**True negatives** (patients without CIDP)	6 studies 135 patients	cohort & case-control type studies	serious[a]	not serious	serious[b]	very serious[c]	strong association	⊕◯◯◯ Very low [a,b,c]
**False positives** (patients incorrectly classified as having CIDP)								

Explanations

a. missing data of control groups, in parts no gender- and age-adjustment, inhomogeneous control groups, heterogeneous study designs

b. variable effect sizes

c. use of different field strengths in some studies while results were not attributable to field strength, data based on few studies with small sample sizes, wide confidence intervals of individual studies

Question: Should contrast enhancement be used to diagnose CIDP in MRI?

Outcome	№ of studies (№ of patients)	Study design	Factors that may decrease certainty of evidence					Test accuracy CoE
			Risk of bias	Indirectness	Inconsistency	Imprecision	Publication bias	
**True positives** (patients with CIDP)	6 studies 92 patients	cohort & case-control type studies	serious[a]	not serious	serious[b]	very serious[c]	strong association	⊕◯◯◯ Very low[a,b,c]
**False negatives** (patients incorrectly classified as not having CIDP)								
**True negatives** (patients without CIDP)	4 studies 46 patients	cohort & case-control type studies	serious[a]	not serious	serious[b]	very serious[c]	strong association	⊕◯◯◯ Very low[a,b,c]
**False positives** (patients incorrectly classified as having CIDP)								

Explanations

a. missing data of control groups, in parts verification bias due to administration of contrast agent only to patients with abnormal baseline MRI

b. highly variable effect sizes of individual studies

c. data based on few studies with small sample sizes, wide confidence intervals of individual studies
