# Supplementary material for: Magnetic Resonance Imaging of Nerve Roots in the Diagnosis of Chronic Inflammatory Demyelinating Polyneuropathy (CIDP) – A Systematic Review and Meta‐Analysis
Source: Eur J Neurol. 2026 May 28;33(5):e70612. doi: 10.1111/ene.70612 (PMC13240206; doi:10.1111/ene.70612)
Supplement: Supplementary file 4 — Figure S4: Funnel plots assessing publication bias of included studies. Funnel plots provided for reference only. [file ENE-33-e70612-s004.pdf]

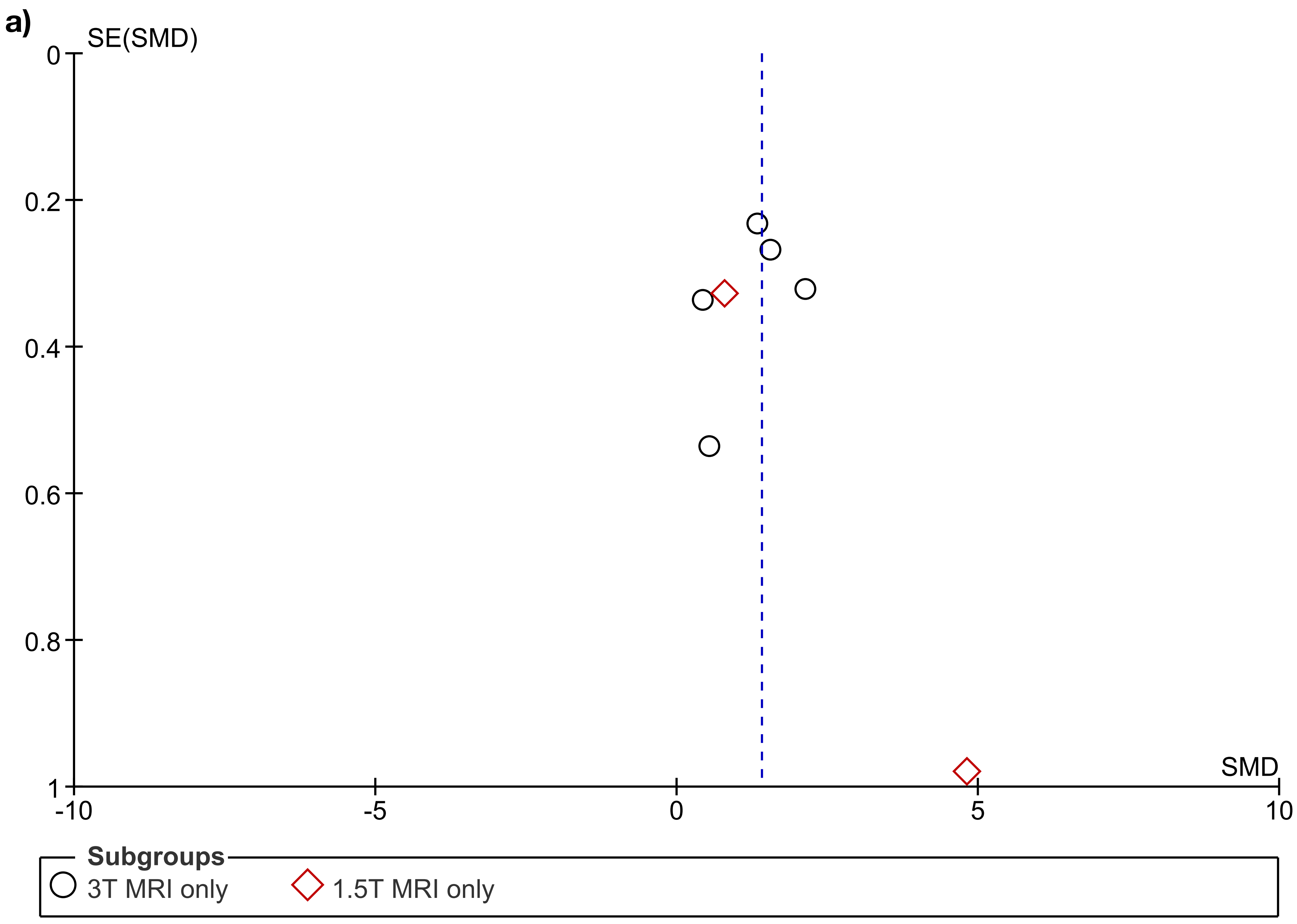

Funnel plot of studies evaluating cervical nerve root diameter on 3T MRI vs. 1.5T MRI in CIDP patients

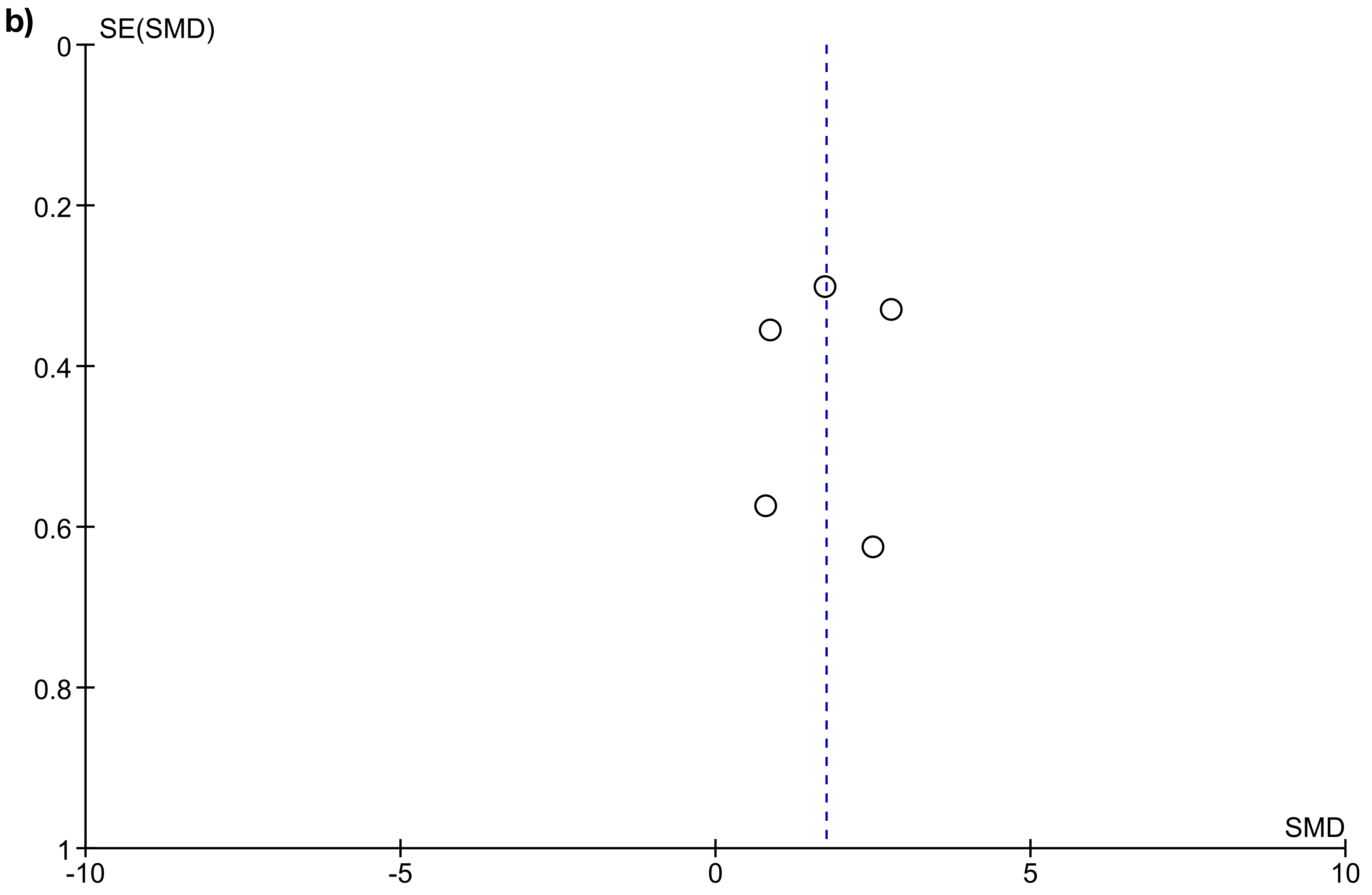

Funnel plot of studies evaluating lumbar nerve root diameter in CIDP patients (all 3T)

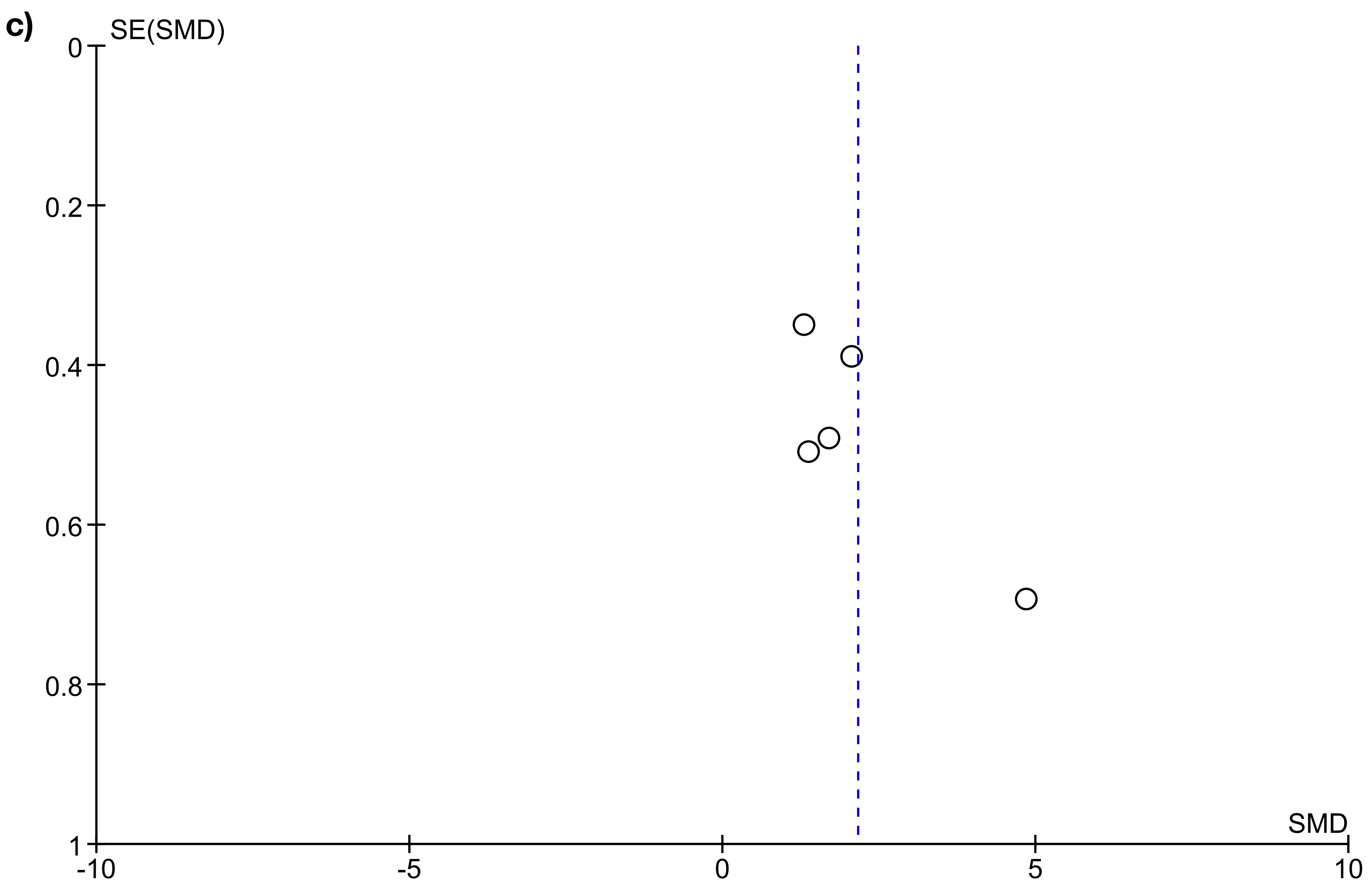

Funnel plot of studies evaluating lumbar cross sectional area (all 3T MRI) in CIDP patients (all 3T)

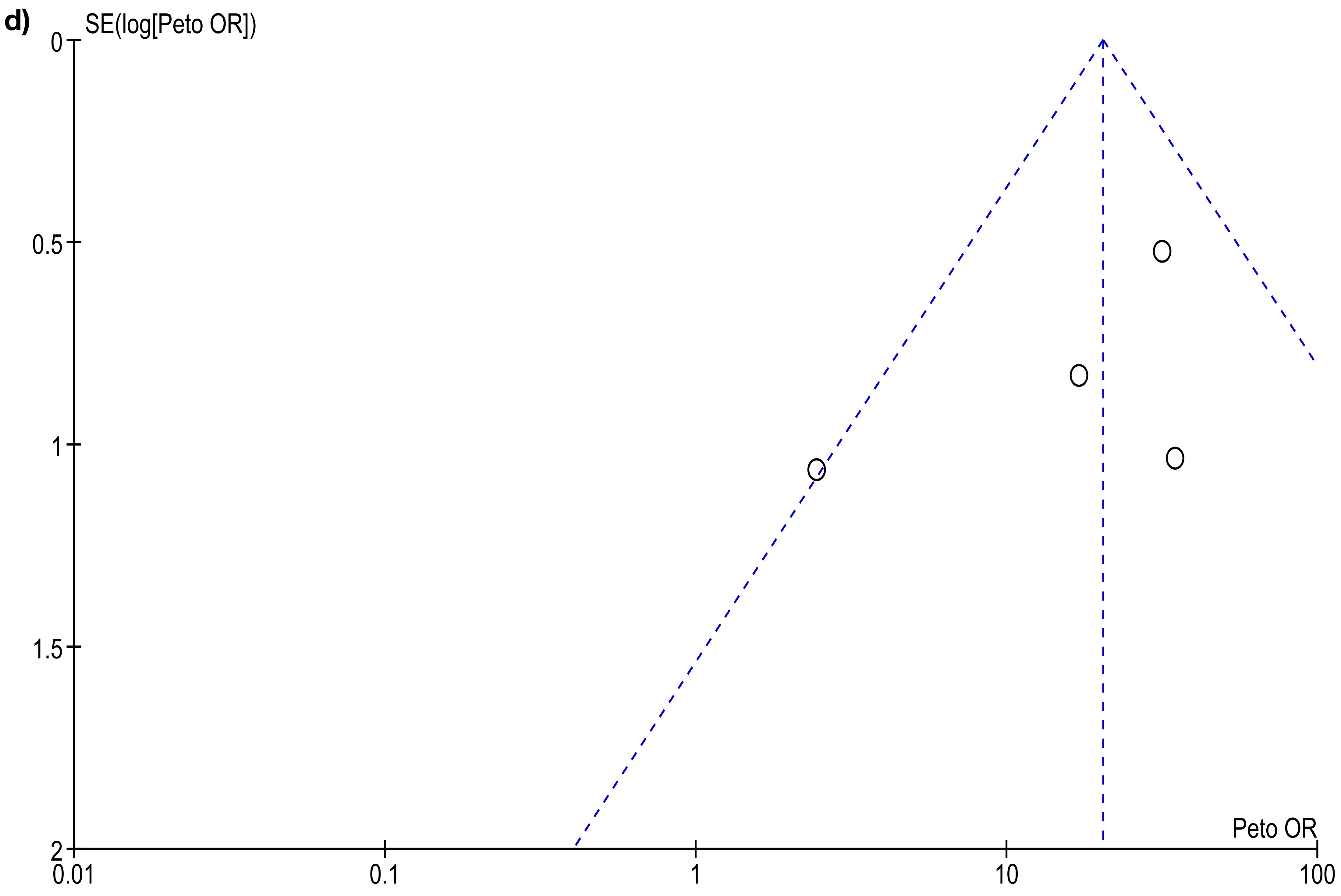

Funnel plot of studies evaluating hypertrophy of nerve roots in CIDP patients

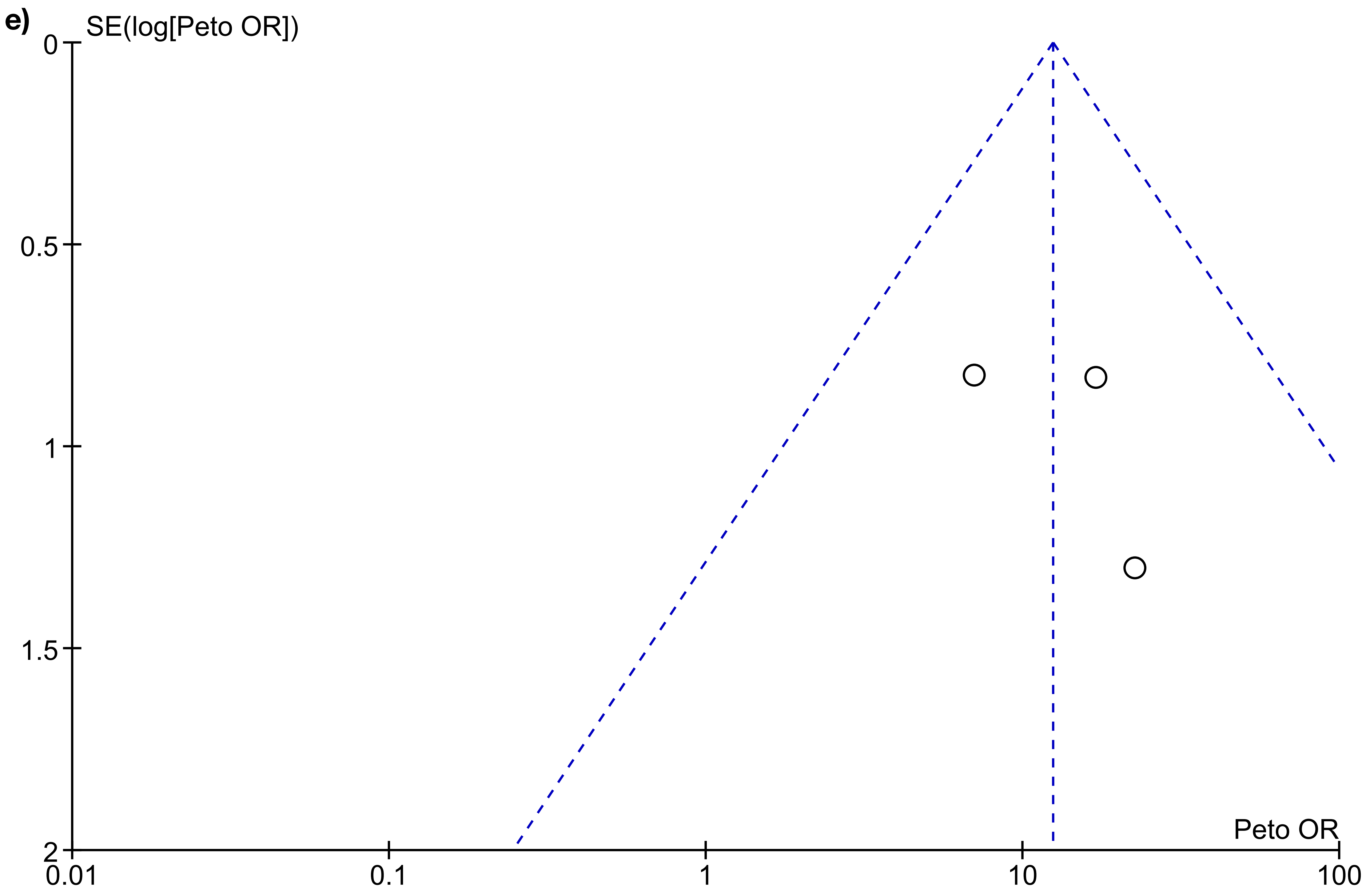

Funnel plot of studies evaluating signal hyperintensity of nerve roots in CIDP patients

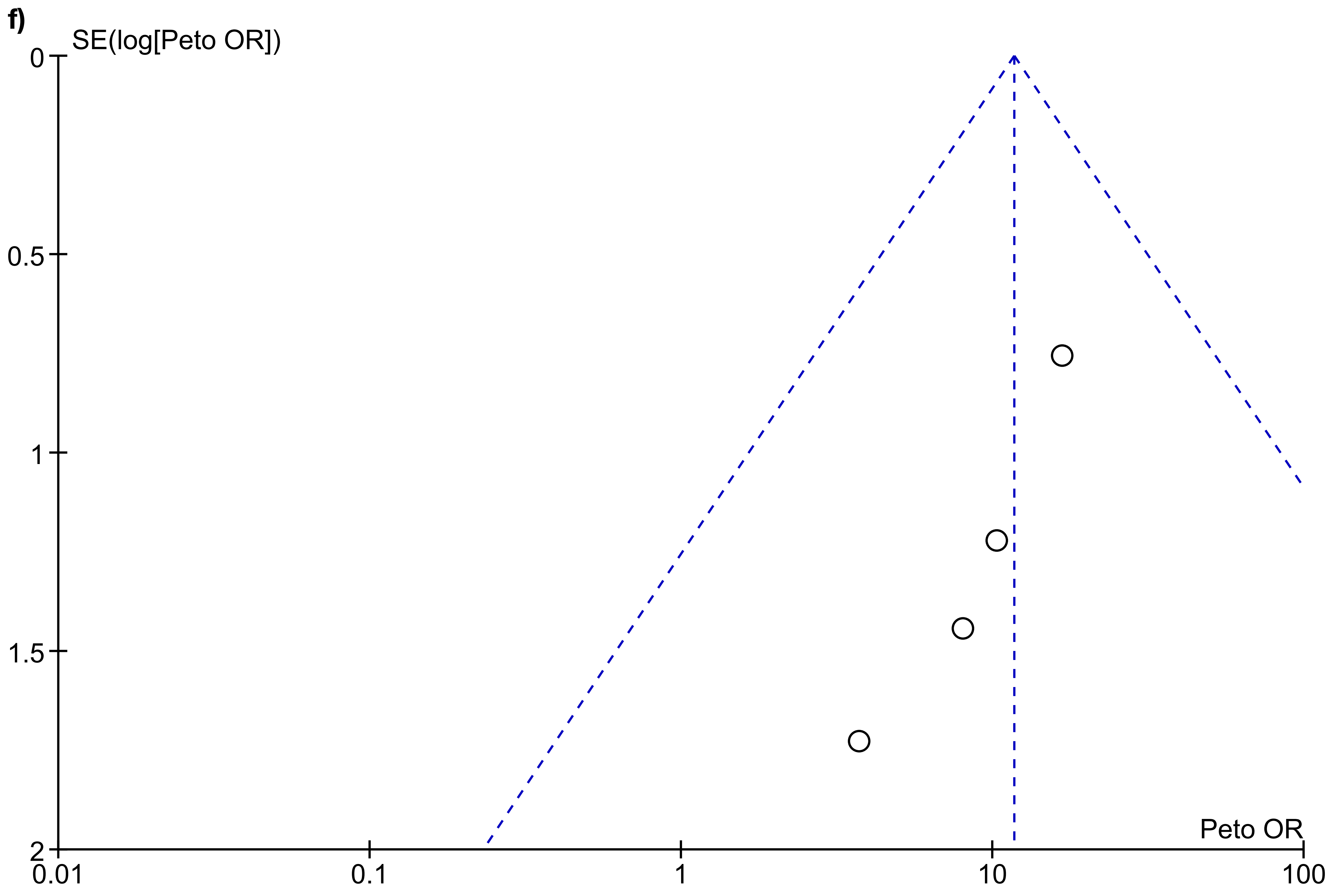

Funnel plot of studies evaluating gadolinium uptake of nerve roots in CIDP patients
